# Ultrasensitive and Multiplexed Protein Imaging with Cleavable Fluorescent Tyramide and Antibody Stripping

**DOI:** 10.3390/ijms22168644

**Published:** 2021-08-11

**Authors:** Thai Pham, Christopher D. Nazaroff, Joshua Labaer, Jia Guo

**Affiliations:** 1 Biodesign Institute & School of Molecular Sciences, Arizona State University, Tempe, AZ 85287, USA; thpham7@asu.edu (T.P.); cnazarof@asu.edu (C.D.N.); Joshua.Labaer@asu.edu (J.L.); 2 Division of Pulmonary Medicine, Department of Biochemistry and Molecular Biology, Mayo Clinic Arizona, Scottsdale, AZ 85259, USA

**Keywords:** immunofluorescence, immunohistochemistry, single-cell, in situ analysis, proteomics, expression heterogeneity, cell–cell interaction

## Abstract

Multiplexed single-cell analysis of proteins in their native cellular contexts holds great promise to reveal the composition, interaction and function of the distinct cell types in complex biological systems. However, the existing multiplexed protein imaging technologies are limited by their detection sensitivity or technical demands. To address these issues, here, we develop an ultrasensitive and multiplexed in situ protein profiling approach by reiterative staining with off-the-shelf antibodies and cleavable fluorescent tyramide (CFT). In each cycle of this approach, the protein targets are recognized by antibodies labeled with horseradish peroxidase, which catalyze the covalent deposition of CFT on or close to the protein targets. After imaging, the fluorophores are chemically cleaved, and the antibodies are stripped. Through continuous cycles of staining, imaging, fluorophore cleavage and antibody stripping, a large number of proteins can be quantified in individual cells in situ. Applying this method, we analyzed 20 different proteins in each of ~67,000 cells in a human formalin-fixed paraffin-embedded (FFPE) tonsil tissue. Based on their unique protein expression profiles and microenvironment, these individual cells are partitioned into different cell clusters. We also explored the cell–cell interactions in the tissue by examining which specific cell clusters are selectively associating or avoiding each other.

## 1. Introduction

Understanding the composition, interaction and regulations of complex biological systems require tools that quantify the abundances of multiple proteins in single cells in their native cellular context [1,2,3]. Mass spectrometry [4] and protein microarray [5] are powerful technologies for comprehensive protein analysis. Nonetheless, these approaches require proteins to be purified and isolated from other cellular components in sample preparation prior to their analysis. Consequently, the protein location information in the biological system is lost. Immunofluorescence is a well-established method for in situ protein profiling. However, as a result of the spectral overlap of the common fluorophores [6], only a handful of different proteins can be visualized by immunofluorescence in one specimen. Imaging matrix-assisted laser deposition/ionization (MALDI) mass spectrometry has been explored for multiplexed in situ protein profiling [7]. Nevertheless, the relatively low imaging resolution hinders its application for single cell analysis. 

To enable multiplexed protein imaging in single cells, a number of methods have been recently developed. In these techniques [8,9,10,11,12,13,14,15,16,17], fluorophores or metal isotopes conjugated to primary antibodies are applied to stain the protein targets. Without signal amplification, the low detection sensitivity of these methods limits their applications to study low-expression proteins or to examine specimens with high autofluorescence, such as formalin-fixed paraffin-embedded (FFPE) tissues [18]. To tackle these issues, several laboratories, including ours, have developed several sensitive and multiplexed protein imaging technologies by signal amplifications with biotin–streptavidin interaction [19], oligonucleotide hybridization [20], and horseradish peroxidase (HRP) [21,22]. However, these methods require a chemical-, oligonucleotide- or HRP-labeled primary antibodies to recognize the protein targets. Such conjugated primary antibodies are usually not commercially available, and to prepare those primary antibodies labeled with the desired tag can be technically demanding, time-consuming and costly. Additionally, these bulky tags on the primary antibodies can interfere with their binding specificity and affinity, leading to false negative and positive staining signals. 

Here, we report a highly sensitive and multiplexed in situ protein profiling approach using cleavable fluorescent tyramide (CFT) and off-the-shelf antibodies. In this approach, protein targets are stained with HRP-conjugated antibodies and CFT. Following image capture, the staining signals are erased by fluorophore cleavage, and HRP is simultaneously deactivated. After all the targets are stained in the first cycle, the antibodies are stripped to initiate the second cycle. Through reiterative cycles of target staining, fluorescence imaging, signal removal and antibody stripping, a large variety of proteins can be quantified in their native spatial contexts at optical resolution. To demonstrate the feasibility of this approach, we show that the microwaving-mediated stripping can efficiently remove the antibodies, and the epitope integrity is maintained for at least 20 analysis cycles. Applying this approach, we quantified 20 different proteins in ~67,000 individual cells in a human FFPE tonsil tissue. Based on their unique protein expression profiles and neighboring cells, these single cells are partitioned into varied cell clusters. By mapping the cell clusters back to their original tissue locations, we observe that different subregions of the tissue are composed of cells from different clusters. We also studied the cell–cell interactions in the tonsil tissue by identifying the association and avoidance among the specific cell clusters. 

## 2. Results

### 2.1. Platform Design

**Figure 1:**
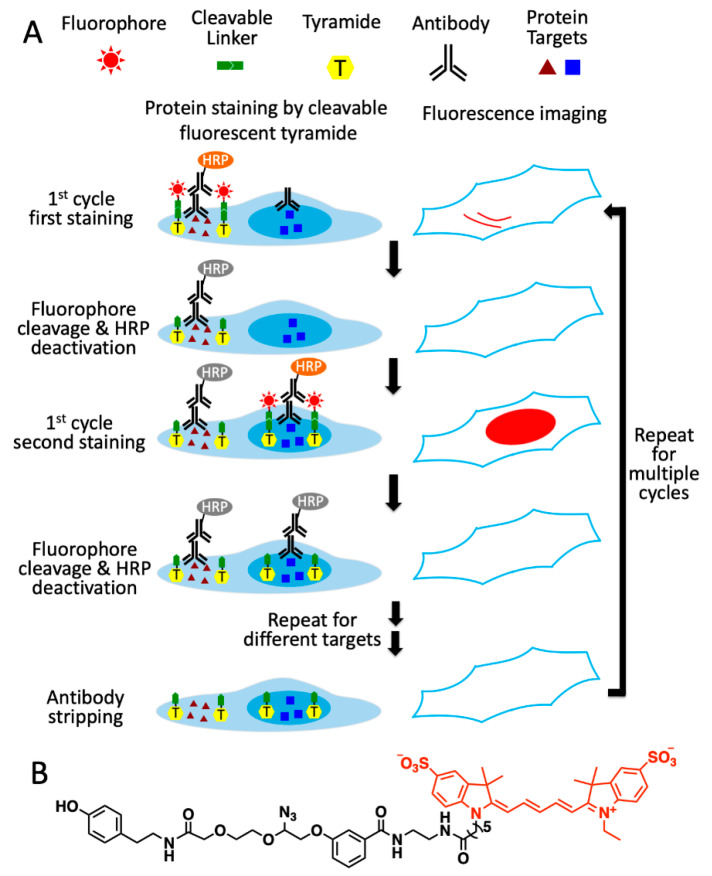
Ultrasensitive and multiplexed protein imaging with cleavable fluorescent tyramide (CFT) and antibody stripping. (**A**) In each cycle, multiple protein targets are first recognized by primary antibodies from different species or of varied immunoglobulin classes. Subsequently, the first target is stained by HRP-conjugated primary or secondary antibodies and CFT. After imaging, the fluorophores are chemically cleaved and HRP is simultaneously deactivated. The processes of protein staining, fluorescence imaging, fluorophore cleavage and HRP deactivation are repeated until every target in the first cycle is stained. Finally, all the antibodies are stripped to initiate the next cycle. Through reiterative analysis cycles, a large number of distinct proteins can be quantitatively profiled in single cells in situ. (**B**) Structure of CFT, tyramide-N_3_-Cy5.

As shown in Figure 1A, this multiplexed in situ protein profiling technology has six major steps in each analysis cycle. First, the different proteins of interest are recognized by primary antibodies from distinct species or of varied immunoglobulin classes. Second, one of protein targets is stained with primary or secondary antibodies conjugated to HRP, which catalyzes the coupling reactions between the tyramide moiety in CFT and the tyrosine residues on the proteins proximal to the target. Third, images of the specimen are captured under a fluorescence microscope to generate quantitative single-cell protein expression profiles. To facilitate the alignment of different protein-staining images, the nucleus stained with DAPI is imaged together with the protein target. Fourth, the fluorophores are cleaved by a chemical reaction, and HRP is simultaneously deactivated. Fifth, steps two to four are repeated until every protein target in the first analysis cycle is quantified. Finally, all the antibodies are stripped to initiate the next cycle. Through reiterative cycles of protein staining, fluorescence imaging, fluorophore cleavage, HRP deactivation and antibody stripping, highly sensitive and multiplexed in situ protein profiling can be achieved in single cells of intact tissues. 

### 2.2. Efficient Antibody Stripping while Preserving Epitope Integrity

**Figure 2:**
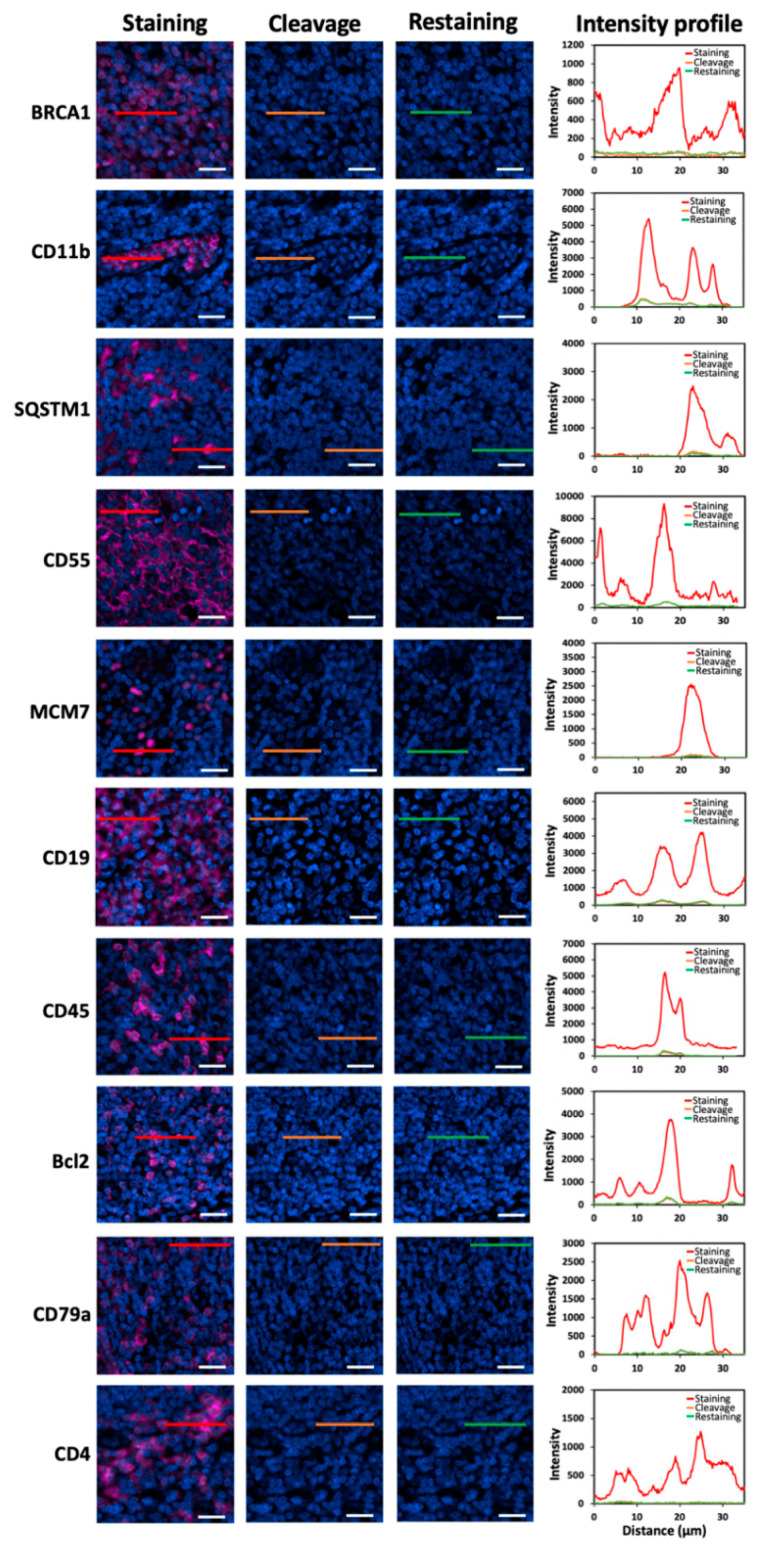

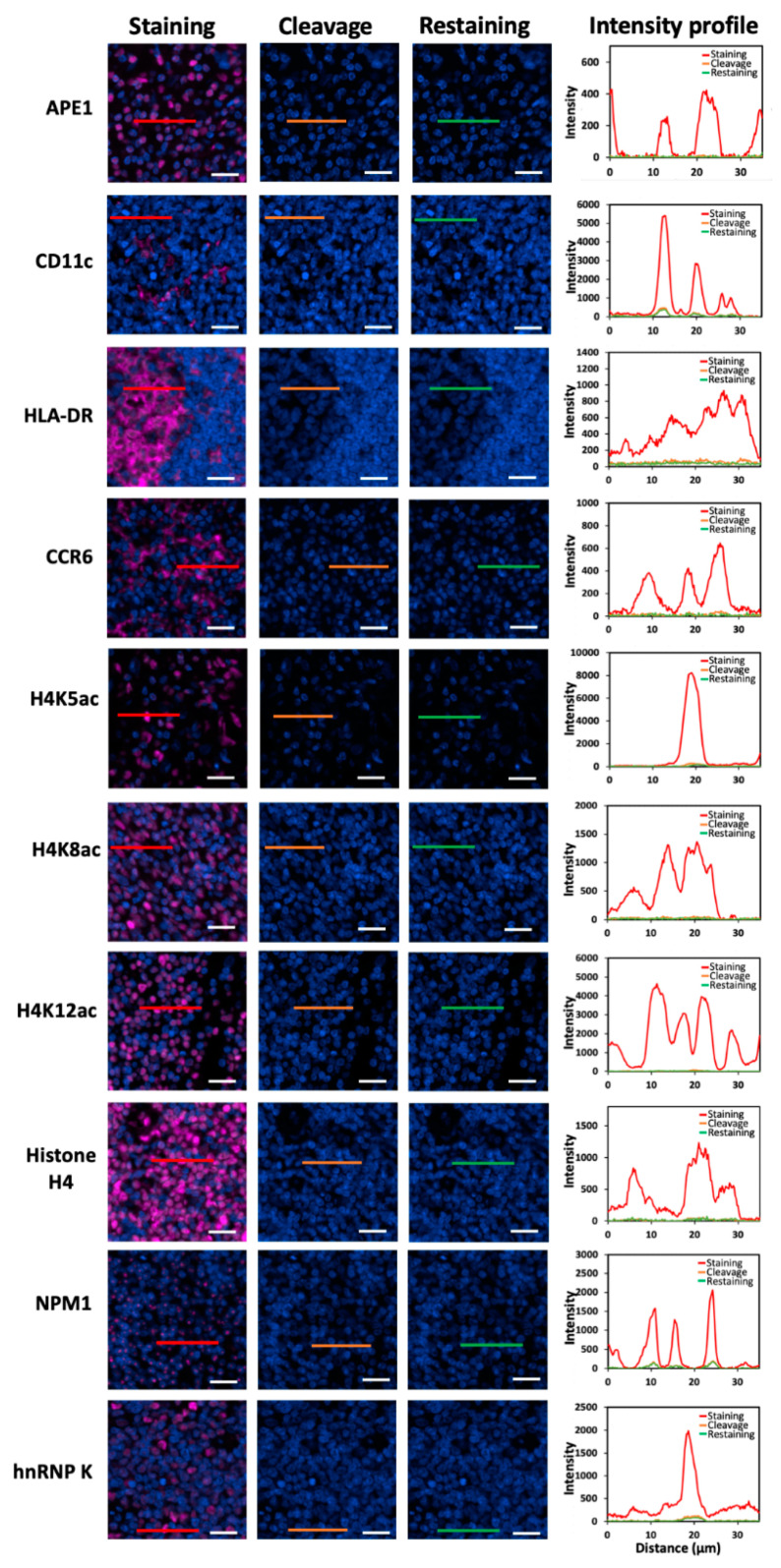
Different proteins are stained with HRP-conjugated antibodies and CFT in FFPE tonsil tissues (the first column). Then, the staining signals are erased by PTA and TCEP (the second column). After antibody stripping, the tissues are re-incubated with HRP-conjugated secondary antibodies and CFT (the third column). The fourth column displays the fluorescence intensity profiles corresponding to the red, orange and green line positions in the first three columns. Scale bars, 20 μm.

One essential requirement for the success of this method is to efficiently strip the antibodies so that the antibodies applied in the previous cycles will not result in false positive signals in the following cycles. To assess the antibody stripping efficiency, we stained 20 different proteins in human FFPE tonsil tissues with HRP-conjugated antibodies and CFT (Figure 2). Subsequently, the fluorophores were cleaved using a mild chemical reaction with 1,3,5-triaza-7-phosphaadamantane (PTA) and tris(2-carboxyethyl)phosphine (TCEP). Following signal removal, almost all the staining signals were erased, confirming the high cleavage efficiency of the CFT as we reported before [22]. We also documented that this chemical reaction does not damage the epitope integrity, which allows other proteins to be accurately profiled in later cycles. Following fluorophore cleavage, the antibodies were removed by microwave-mediated stripping. Afterwards, the tissues were re-incubated with HRP-conjugated antibodies and CFT. However, no signal increase was observed. These results indicate that the antibodies applied for protein staining can be efficiently removed by microwave-mediated stripping.

**Figure 3:**
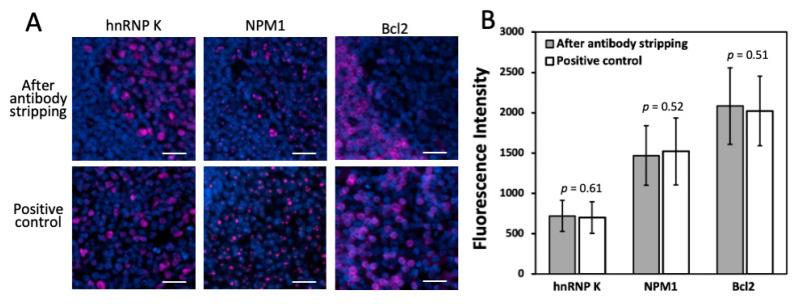
(**A**) Protein hnRNP K, NPM1 and Bcl2 are stained with CFT in the same FFPE tonsil tissue after 10, 15 and 20 cycles of antibody stripping, respectively (top row). These proteins are also stained in three different FFPE tonsil tissues by the conventional tyramide signal amplification approach (bottom row). (**B**) Comparison of the staining intensities obtained after antibody stripping and by conventional immunofluorescence (*n* = 50 positions). Error bars, standard deviation. Scale bars, 20 μm.

Another critical requirement for this approach to succeed is that the epitope integrity must be maintained under this antibody stripping condition. In this way, the stripping process applied in the prior cycles will not interfere with the precise protein profiling in the later cycles. To evaluate the effects of antibody stripping on epitope integrity, we stained proteins hnRNP K, nucleophosmin and Bcl2 in the same human FFPE tonsil tissue, after 10, 15 and 20 cycles of antibody stripping, respectively (Figure 3A). As positive controls, we also stained the same three proteins in three FFPE tonsil tissues using the conventional tyramide signal amplification approach. The staining patterns and signal intensities (Figure 3B) obtained by the two methods are consistent with each other. These results suggest that the epitope integrity is preserved after at least 20 cycles of antibody stripping.

### 2.3. Multiplexed In Situ Protein Profiling in FFPE Tissues

**Figure 4:**
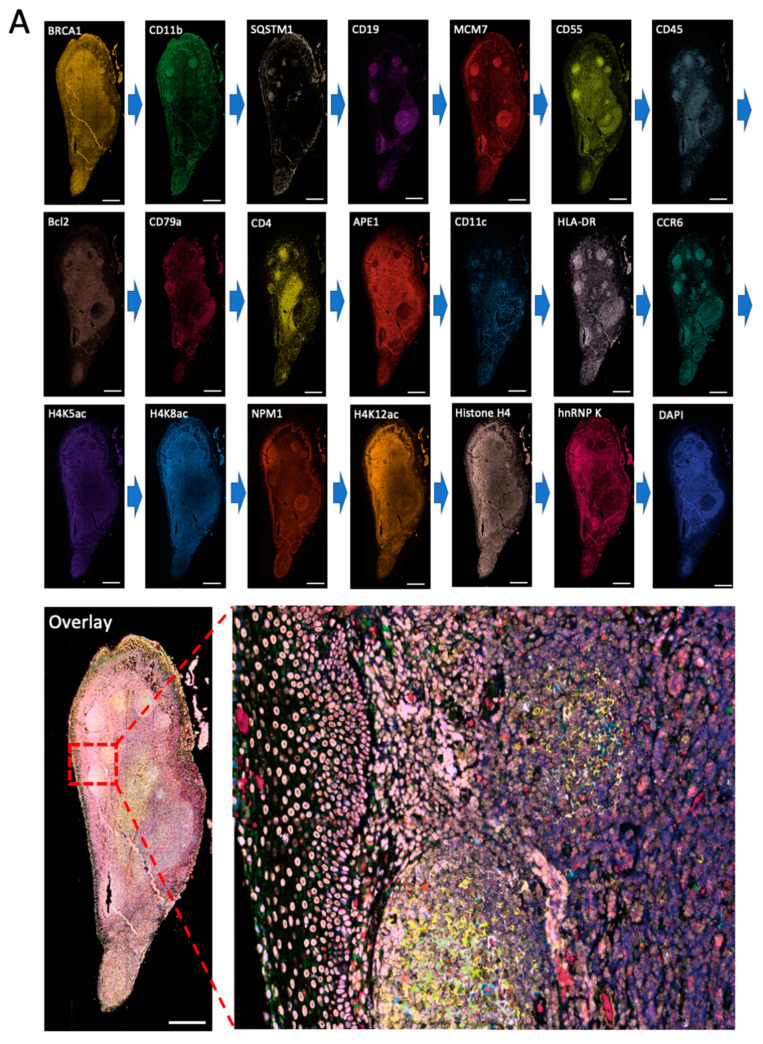

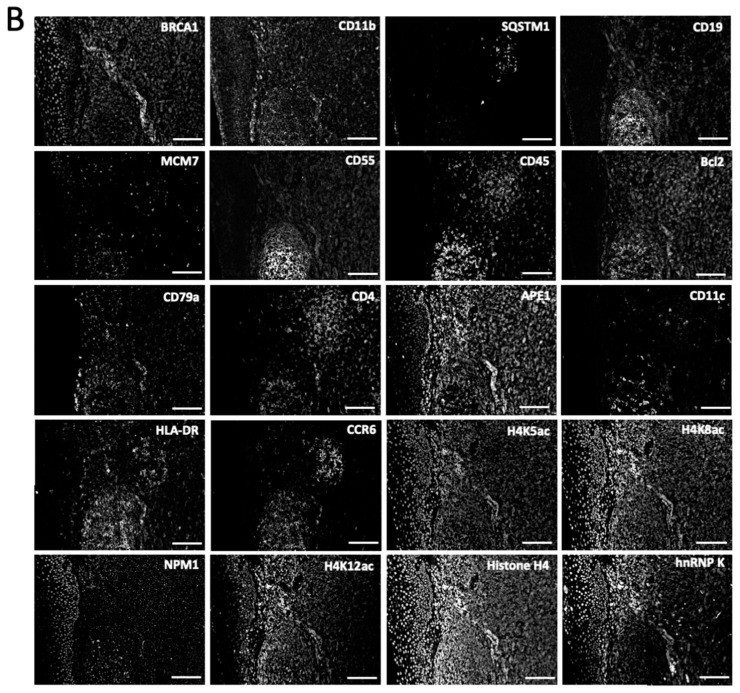
(**A**) The 20 different proteins are stained with CFT in the same FFPE tonsil tissue. Scale bars, 500 μm. (**B**) Zoomed-in views of the boxed area in (**A**). Scale bars, 100 μm.

To demonstrate the feasibility of applying this approach for multiplexed in situ protein profiling in FFPE tissues, we stained 20 different proteins using off-the-shelf HRP-conjugated antibodies and CFT in the same FFPE human tonsil tissue (Figure 4). All 20 proteins were successfully stained and unambiguously detected at subcellular resolution. The obtained protein staining patterns are consistent with the ones generated by staining each protein in different tissues (Figure 2). Due to its high detection sensitivity resulting from the HRP signal amplification, our approach allows the imaging time to be dramatically reduced while maintaining the analysis accuracy. By automatic whole slide scanning with the fluorescence microscope, it only takes less than 10 min to image this tissue (~2 mm × 4 mm). In comparison, the current mass spectrometry imaging methods require ~64 h to image tissue of similar sizes [1]. These results indicate that our approach enables highly sensitive and multiplexed in situ protein profiling in FFPE tissues with short assay time and high sample throughput. 

### 2.4. Different Cell Types and Their Spatial Distribution in the Human Tonsil Tissue

**Figure 5:**
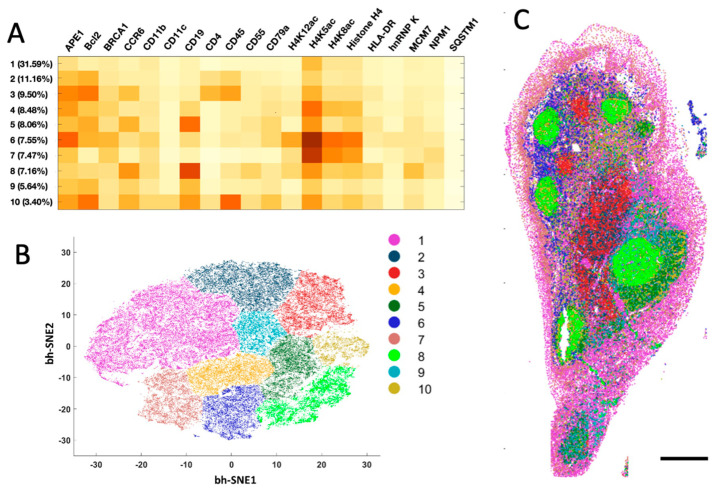
(**A**) Based on their different single cell protein expression profiles, (**B**) ~67,000 individual cells in the human tonsil tissue are partitioned into 10 cell clusters. (**C**) Anatomical locations of each cell from the 10 clusters. Scale bar, 500 μm.

The generated single-cell in situ protein expression profiles also allow us to study cell heterogeneity and the spatial distribution of the various cell types in human tonsil tissues. To achieve that, we calculated the expression levels of the 20 examined proteins in each of ~67,000 cells identified in the tissue. Based on their unique protein expression patterns (Figure 5A and Supplementary Figure S1), those individual cells were partitioned into 10 different cell clusters (Figure 5B) using the software viSNE [23]. We then mapped these 10 cell clusters back to their natural tissue locations (Figure 5C and Supplementary Figure S2) and observed that the varied subregions of tonsil tissue are composed of cells from distinct clusters. For instance, cluster 7 is the major cell type in epithelium. The germinal centers mainly consist of clusters 8 and 10, while the lymph nodules are dominated by clusters 5, 6 and 9. Clusters 1 and 4 only appear in connective tissues. These results indicate that our approach enables the study of cell-type classification and their spatial distribution in FFPE tissues.

### 2.5. Cell–Cell Interactions in the Human Tonsil Tissue

**Figure 6:**
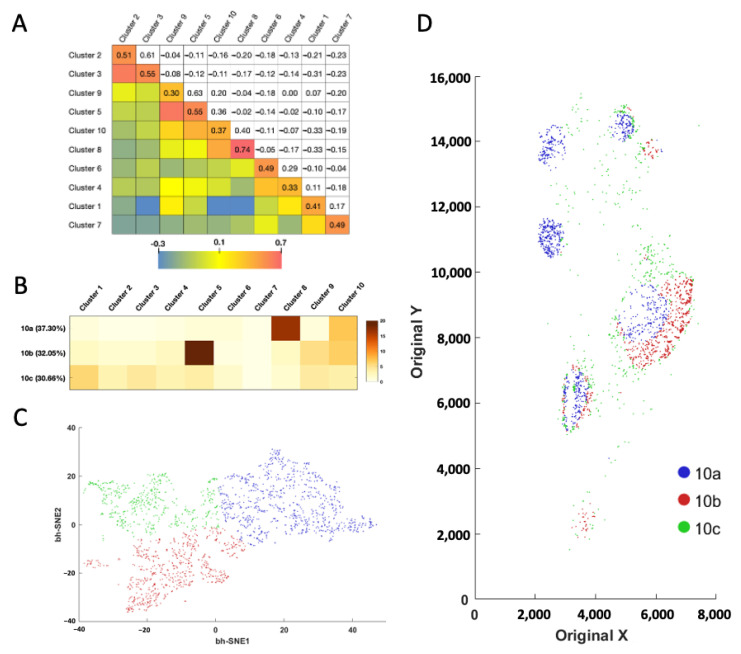
(**A**) Cell cluster to cell cluster interaction strength heatmap. The upper triangle shows the correlation coefficient of the cell numbers in the identified cell neighborhoods. The lower triangle displays the color corresponding to the correlation coefficient. (**B**) Based on their neighbor cells from different clusters, (**C**) the individual cells in cluster 10 are further partitioned into three subclusters. (**D**) Anatomical locations of each cell from the three subclusters.

With the proteins profiled at their native spatial contexts, our approach also allows the investigation of cell–cell contacts between different cell clusters (Figure 6A). To achieve that, we defined the cell neighborhood as all the cells within the 20 μm distance of a central cell. For the ~67,000 individual central cells in the human tonsil tissue, we counted the cell number from varied clusters in each of the cell neighborhoods. We then calculated the correlation coefficient of those cell numbers between each pair of the different cell clusters. When displayed in the heatmap, the metrics revealed that some cell clusters are selectively associated with or avoiding each other. For example, a significant association was observed between cell clusters 2 and 3, 5 and 9, together with 8 and 10, while an avoidance of contact was revealed between cell clusters 1 and 3, 1 and 8, along with 1 and 10. Interestingly, we observed a consistently strong association between cells from same cell cluster (Figure 6A, diagonal), indicating that homotypic cell adhesion may play an important role in formation of the architecture of human tonsil tissue. 

Based on the cell number from the distinct clusters in their neighborhoods (Figure 6B and Supplementary Figure S3), the single cells in the same cluster can be partitioned into subclusters (Figure 6C and Supplementary Figure S3). By mapping these newly identified cell subclusters back to their native tissue locations, we observed the different subclusters from the same cell cluster are located at varied subregions of the tonsil tissue (Supplementary Figure S3). For example, subcluster 10a mainly appears in the germinal centers, the majority of subcluster 10b is observed at the lymph nodules, while almost all the subcluster 10c is located in connective tissues (Figure 6D). These results suggest that our approach allows the investigation of cell–cell contact and cell-type classification based on the microenvironment. 

## 3. Discussion

In this study, we have demonstrated that ultrasensitive and multiplexed in situ protein profiling can be successfully achieved in single cells of FFPE tissues using CFT and antibody stripping. In comparison with the current multiplexed protein imaging technologies, our approach has ultrahigh detection sensitivity by the enzymatic signal amplification. As a result, low-expression proteins or the targets in highly autofluorescent tissues can be accurately quantified. In addition, by eliminating the requirement for the sophisticated chemical conjugation of primary antibodies, the binding specificity and affinity of the antibodies are maintained. Moreover, by enabling the large number of the commercially available unconjugated primary antibodies to be directly applied in the assay, our approach can be easily adopted by research laboratories and in clinical settings. 

Applying this method, we have shown that the individual cells in human tonsil tissue can be classified into different cell clusters based on their unique multiplexed protein expression profiles, and the varied subregions of the tonsil tissue consist of cells from different clusters. We also explored which cell clusters are associating or avoiding each other. Depending on the distinct cell clusters of their neighbor cells, the single cells in each cluster are further partitioned into varied subclusters. These results suggest that our approach allows the classification of cell types and subtypes based on their protein expression profiles and neighbor cells. These identified cell types and subtypes will bring new insights into cell heterogeneity studies, disease diagnosis, and patient stratification. 

The multiplexing capacity of this protein imaging technology depends on the cycling number and the number of proteins interrogated in each analysis cycle. Here we demonstrated that the integrity of the protein epitopes is maintained after at least 20 times of protein stripping. Recently, we also reported that the PTA and TCEP treatment does not damage the epitopes [22]. These results suggest more than 20 analysis cycles can be performed on one tissue sample. In each cycle, the protein targets can be stained with primary antibodies from different species or of varied immunoglobulin classes, hapten or HRP-conjugated primary antibodies. With four or five CFT consisting of distinct fluorophores applied in each cycle, together with repeated protein staining and fluorophore cleavage, potentially up to 10 protein targets can be quantified in one analysis cycle. As a result, we envision that this multiplexed protein imaging method has the potential to profile hundreds of varied protein targets in the same specimen. 

This in situ protein analysis method can also be combined with nucleic acids [24,25,26,27,28,29,30,31,32] and metabolic imaging technologies [33] to enable the integrated DNA, RNA, protein and metabolic profiling of single cells in intact tissues. Moreover, a program-controlled microfluidic system [34] together with a standard fluorescence microscope can be easily made into an automatic tissue imaging platform. This highly multiplexed molecular imaging system would have wide applications in systems biology and biomedical studies. 

## 4. Materials and Methods

### 4.1. General Information

Chemicals and solvents were purchased from Sigma-Aldrich (St. Louis, MO, USA) or TCI America (Portland, OR, USA) and were used directly without further purification. Bioreagents were purchased from Abcam (Cambridge, United Kingdom), Invitrogen (Waltham, MA, USA), or Novus Biologicals (Littleton, CO, USA), unless otherwise noted.

### 4.2. Deparaffinization and Antigen Retrieval of FFPE Tonsil Tissue

After heated at 60 °C for 1 h, tonsil FFPE tissue slides (NBP2-30207, Novus Biologicals (Littleton, CO, USA)) were deparaffinized in xylene three times, each for 10 min. The slide was then immersed successively in 50/50 xylene/ethanol for 2 min, 100% ethanol for 2 min, 95% ethanol for 2 min, and 70% ethanol for 2 min. The slides were rinsed with deionized water. Thereafter, heat induced antigen retrieval (HIAR) was performed using a microwave. The slide was immersed in antigen retrieval citrate buffer (Abcam ab64236) and heated in the microwave for 2 min and 45 s at high power (level 10) and 14 min at low power (level 2). After cooling to room temperature for 20 min, the slide was incubated with 3% H_2_O_2_ in PBT (0.1% Triton-X 100 in 1X phosphate buffer saline (PBS)) to deactivate endogenous horse radish peroxidase (HRP) for 10 min. Subsequently, the slide was washed with PBT for 5 min twice before proceeding to Immunofluorescence with CFT.

### 4.3. Protein Staining in FFPE Tonsil Tissue 

The slides were incubated with antibody blocking buffer (0.1% (vol/vol) Triton X-100, 1% (wt/vol) bovine serum albumin and 10% (vol/vol) normal goat serum) at room temperature for 30 min. Subsequently, the slides were incubated with 5 μg/mL of primary antibody (Table 1) in antibody blocking buffer for 1 h, followed by 3 times 5 min washes with PBT. Then, the slides were incubated with 10 μg/mL of goat anti-rabbit, HRP or goat anti-mouse, HRP (Table 1) in antibody blocking buffer for 1 h, and then washed 3 times with PBT, each for 5 min. Afterwards, the slide were stained with tyramide-N_3_-Cy5 at the concentration of 10 nmol/mL in amplification buffer (0.003% H_2_O_2_, 0.1% Tween-20, in 100 mM borate, pH = 8.5) for 10 min at room temperature, and then washed twice with PBT, each for 5 min. The tissues were stained with DAPI and mounted with Prolong Diamond Antifade Mountant before proceeding to imaging. 

### 4.4. Fluorophore Cleavage and HRP Deactivation

The stained tissues were incubated with 100 mM 1,3,5-triaza-7-phosphaadamantane (PTA) and 100 mM tris(2-carboxyethyl)phosphine (TCEP) for 30 min sequentially at 40 °C. Subsequently, the slides were washed 3 times with PBT and 1X PBS, each for 5 min.

### 4.5. Antibody Stripping

The slides were immersed in antigen retrieval citrate buffer (Abcam ab64236) and heated in the microwave for 2 min and 45 s at high power (700 watt, level 10) and 14 min at low power (140 watt, level 2). Then, the tissues were cooled down to room temperature for 20 min. 

### 4.6. Imaging and Data Analysis

FFPE Tonsil tissues were imaged under a Nikon Ti-E epifluorescence microscope equipped with a 20× objective. Images were captured using a CoolSNAP HQ2 camera and C-FL DAPI HC HISN together with Chroma 49009 filters. Image data was processed with NIS-Elements Imaging software. The DAPI images in every cycle were used as coordination reference for aligning the images from different cycles. For the single-cell protein expression profiles, cells were segmented based on nuclear staining by DAPI using NIS-Elements Imaging software. The DAPI signals were expanded by 10 pixels for every cell to determine the regions of interest (ROIs). The signal intensity values within the ROIs of each cell were calculated using CellProfiler [35], and the resulting single-cell signal intensity profiles were converted into comma separated value (CSV) files. Then, these files were used for unsupervised clustering by CYT to generate ViSNE plots (https://www.c2b2.columbia.edu/danapeerlab/html/cyt.html) (5 January 2021) [23]. All pseudo-color images were generated with ImageJ. Cell neighborhoods were calculated by detecting and classifying the surrounding cells within 20 μm or less of each individual cell in the sample. The number of cells from the different clusters in each cell neighborhood were used for clustering by CYT to generate subcluster ViSNE plots.

## Figures and Tables

**Table 1:** The antibodies used in this study.

No.	Antibody Target	Catalog Number	Host	Conjugation	Source
1	CD4	133,616	Rabbit	None	Abcam
2	CCR6	227,036	Rabbit	None	Abcam
3	HLA-DR	20,181	Mouse	None	Abcam
4	APE1	194	Mouse	None	Abcam
5	SQSTM1	56,416	Mouse	None	Abcam
6	CD11b	224,805	Rabbit	None	Abcam
7	CD11c	52,632	Rabbit	None	Abcam
8	CD19	134,114	Rabbit	None	Abcam
9	Bcl-2	182,858	Rabbit	None	Abcam
10	CD79a	199,001	Mouse	None	Abcam
11	BCRA1	16,780	Mouse	None	Abcam
12	H4K8ac	45,166	Rabbit	None	Abcam
13	H4K5ac	51,997	Rabbit	None	Abcam
14	H4K12ac	177,793	Rabbit	None	Abcam
15	Histone H4	177,840	Rabbit	None	Abcam
16	MCM7	2360	Mouse	None	Abcam
17	NPM1	202,579	Mouse	HRP	Abcam
18	hnRNP K	204,456	Mouse	HRP	Abcam
19	CD55	133,684	Rabbit	None	Abcam
20	CD45	187,281	Rabbit	None	Abcam
21	Rabbit IgG	6721	Goat	HRP	Abcam
22	Mouse IgG	6789	Goat	HRP	Abcam

## Data Availability

Not applicable.

## Supplementary Materials

The following are available online at https://www.mdpi.com/article/10.3390/ijms22168644/s1.
