# Supplementary material for: Ultrasensitive and Multiplexed Protein Imaging with Cleavable Fluorescent Tyramide and Antibody Stripping
Source: Int J Mol Sci. 2021 Aug 11;22(16):8644. doi: 10.3390/ijms22168644 (PMC8395465; doi:10.3390/ijms22168644)
Supplement: Supplementary file 1 [file ijms-22-08644-s001.zip › ijms-1322374-supplementary.pdf]

Supplementary Material

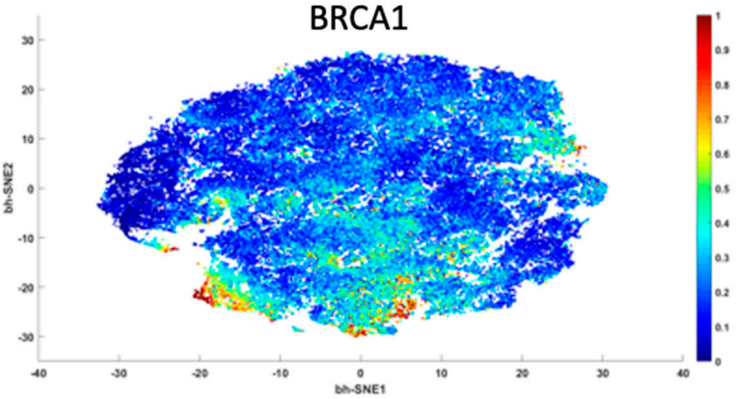

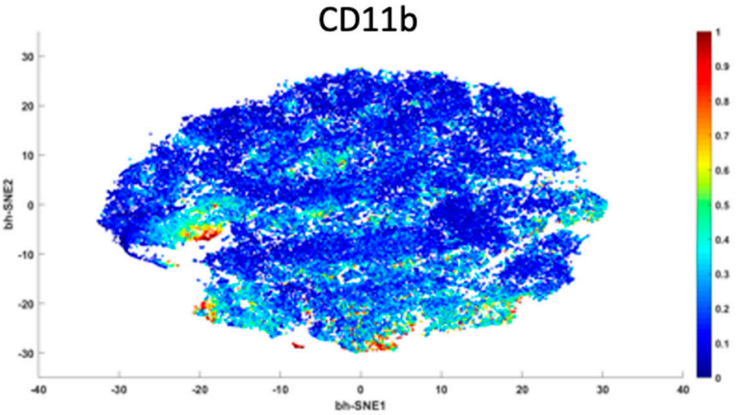

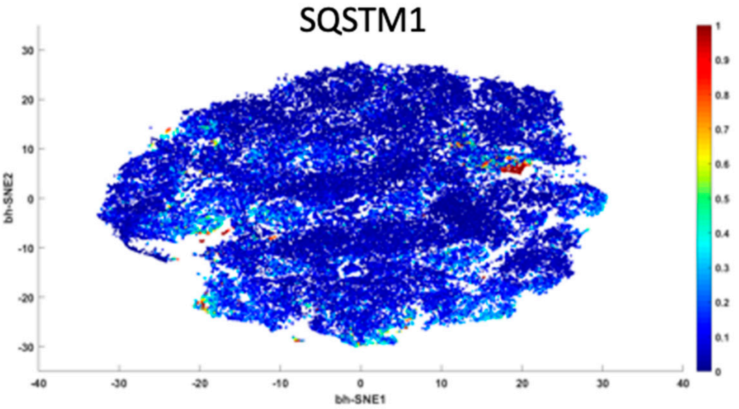

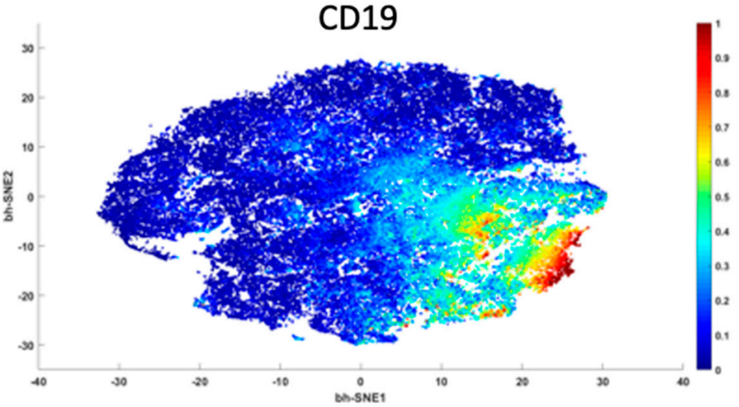

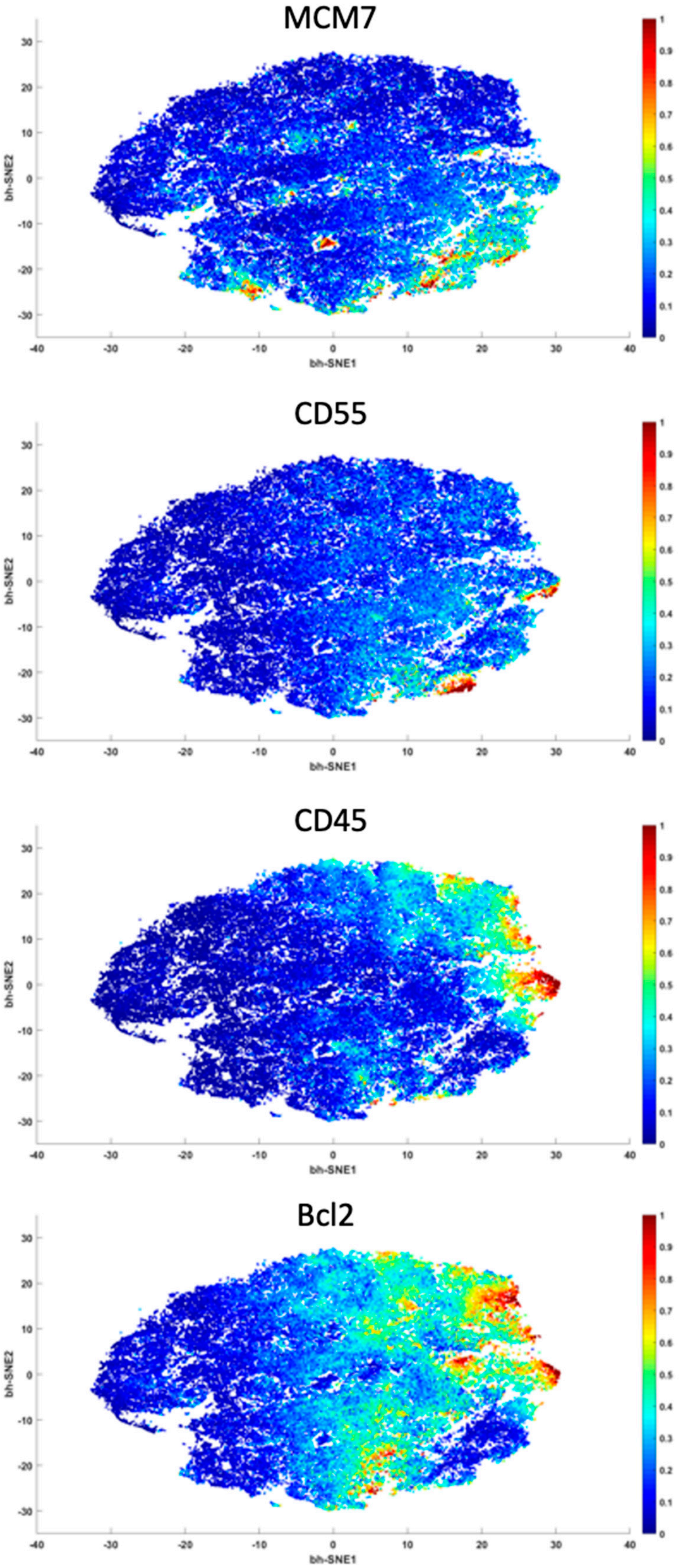
MCM7
CD55
CD45
Bcl2
bh-SNE1
bh-SNE2

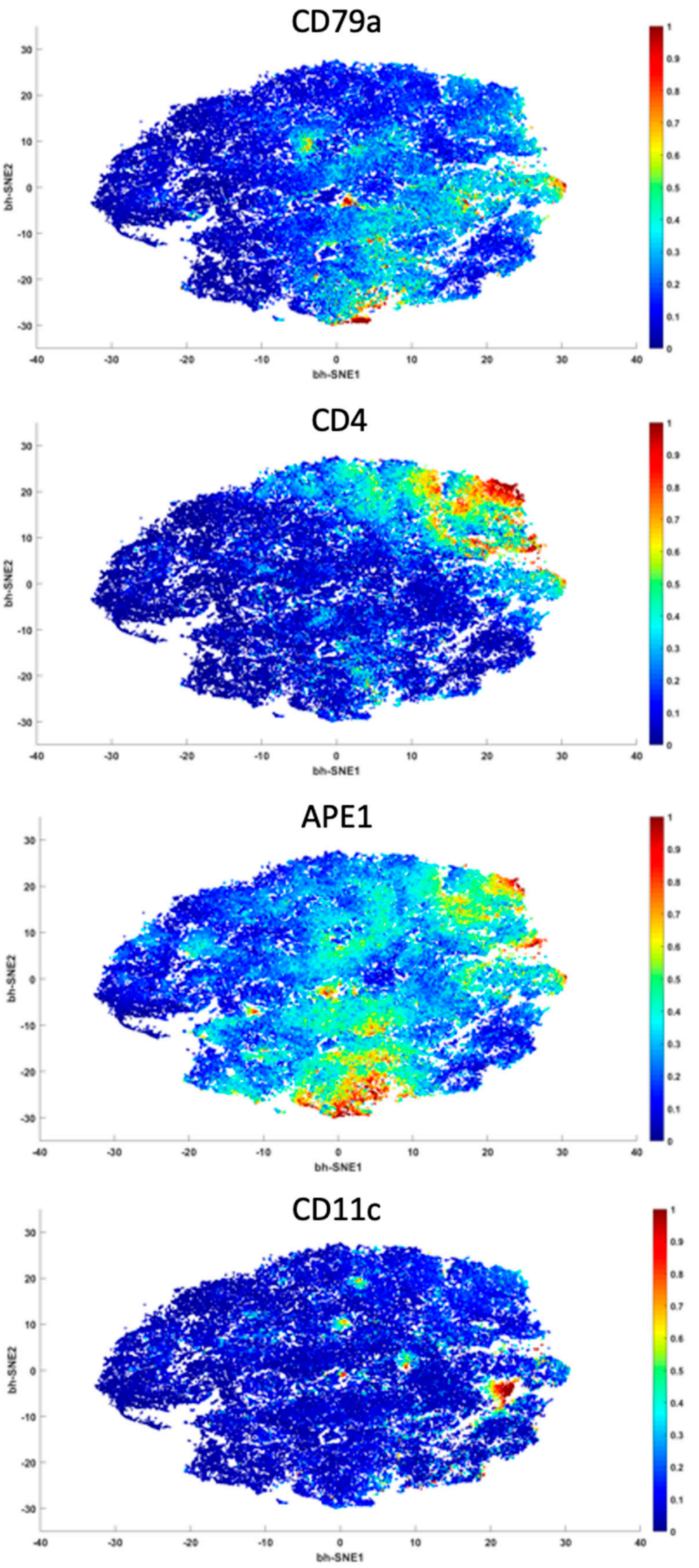

CD79a
CD4
APE1
CD11c
bh-SNE1
bh-SNE2

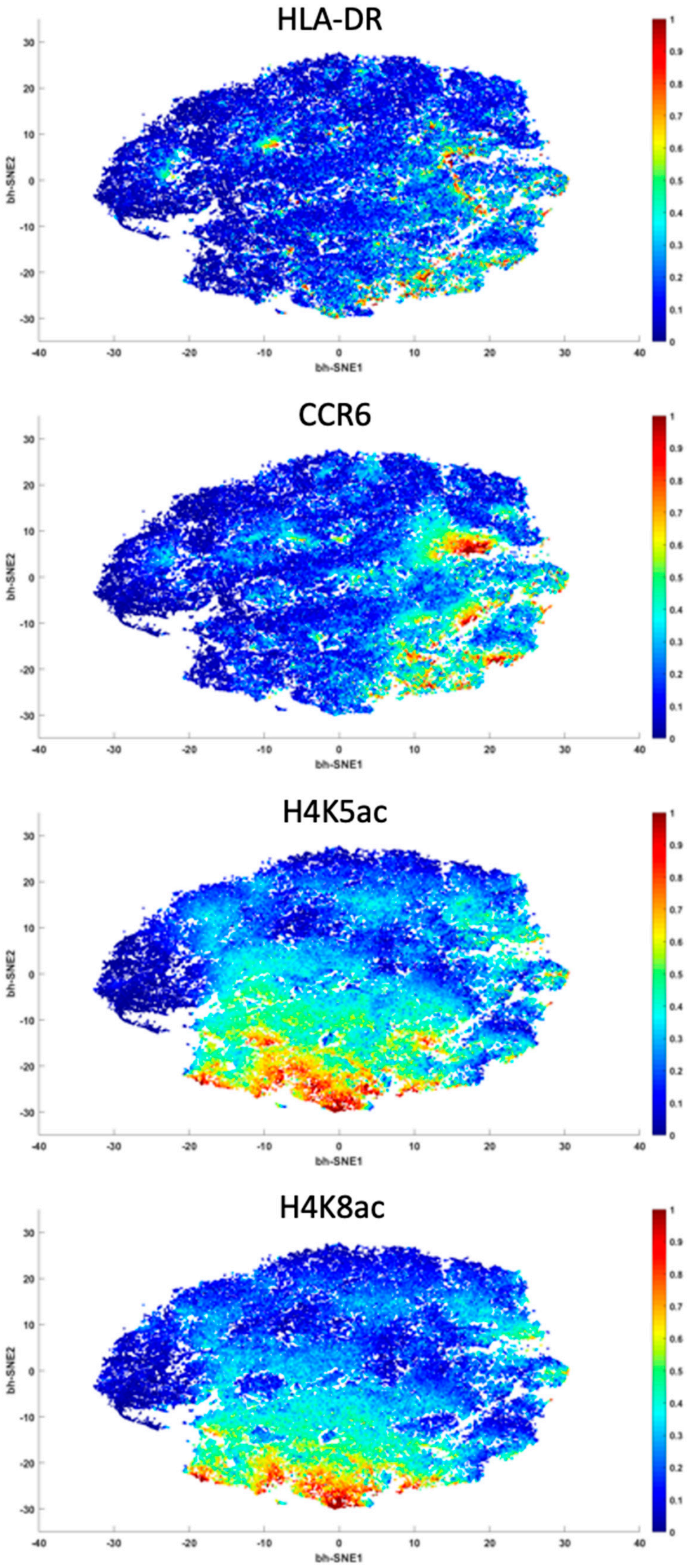
HLA-DR
bh-SNE2
bh-SNE1
CCR6
bh-SNE2
bh-SNE1
H4K5ac
bh-SNE2
bh-SNE1
H4K8ac
bh-SNE2
bh-SNE1

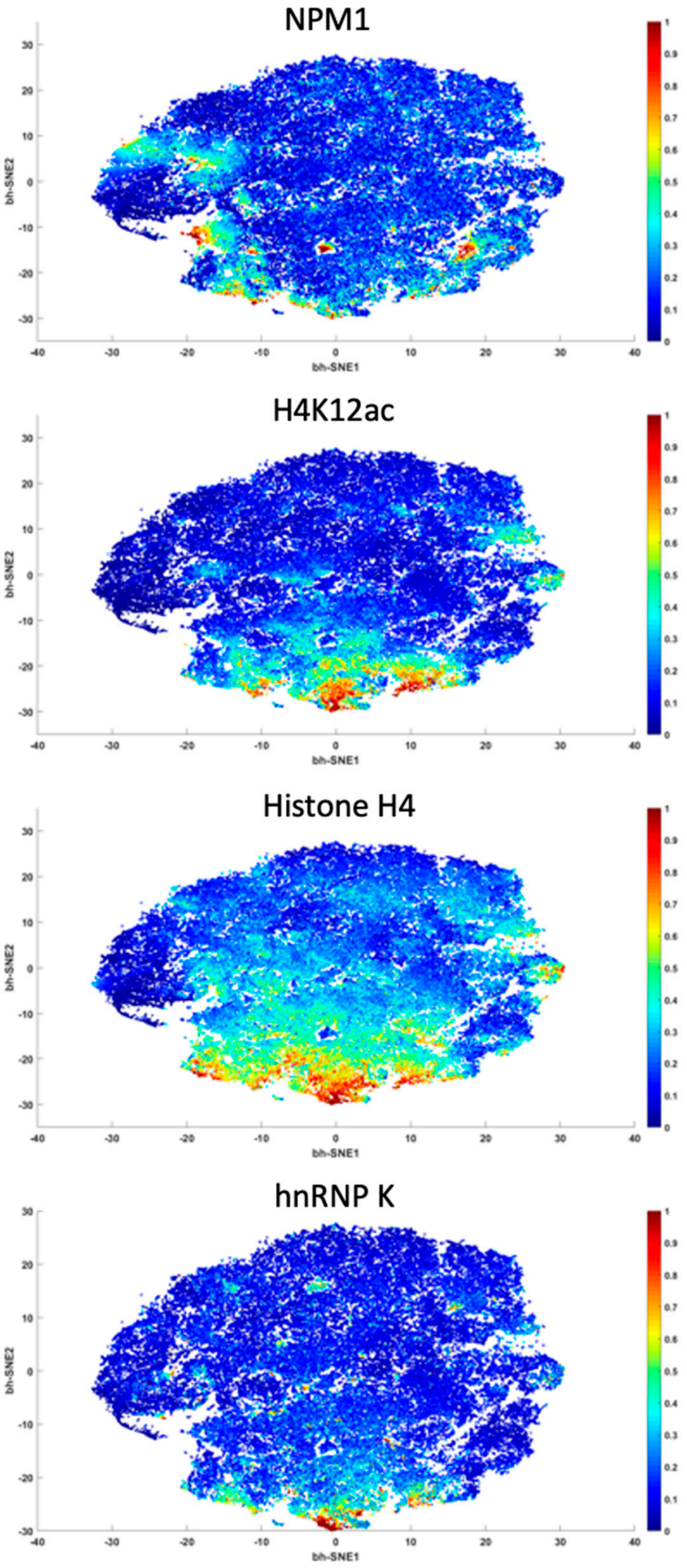

Figure S1. Single cell protein expression distribution in viSNE plots.

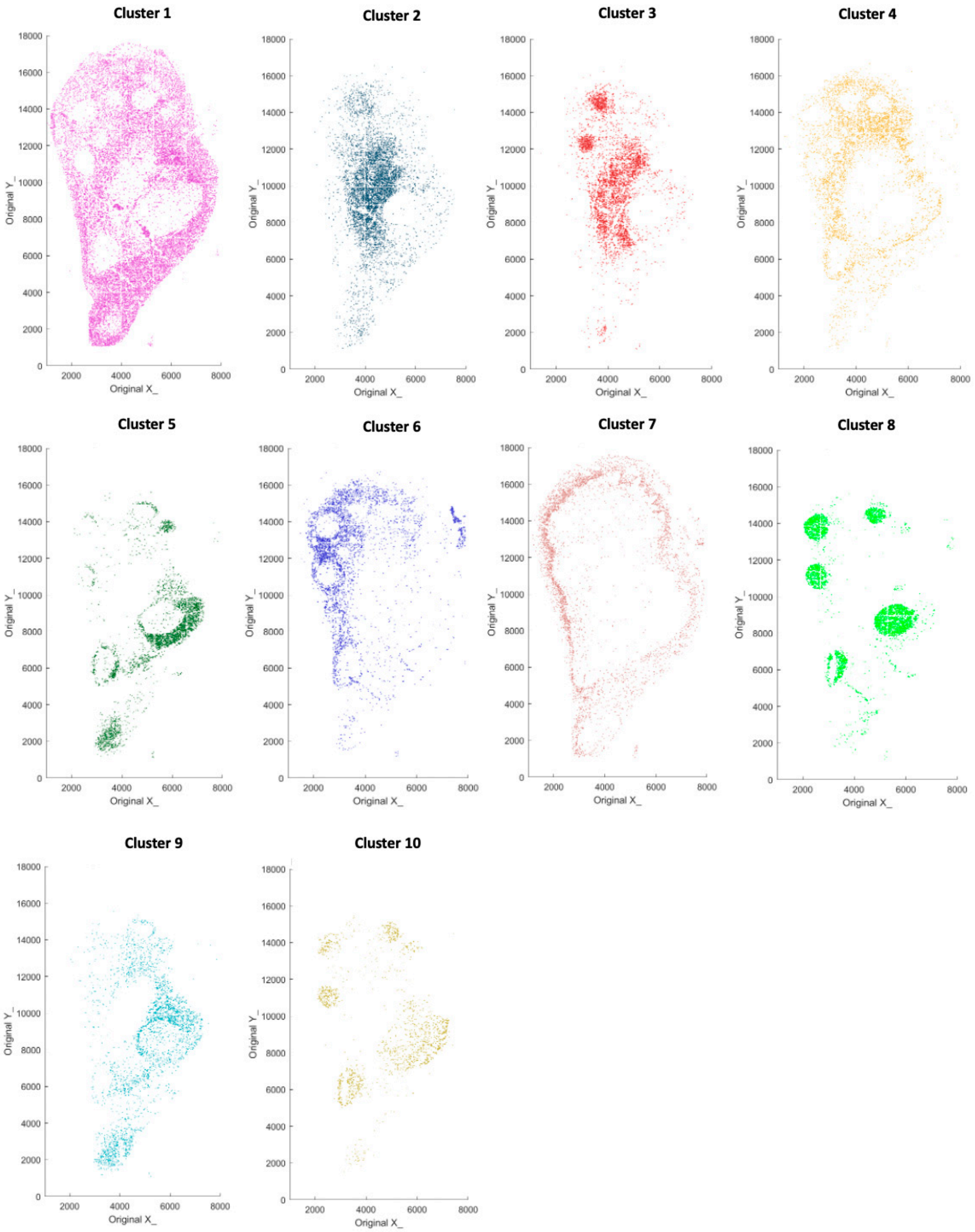

Figure S2. Anatomical locations of the individual cells from the 10 different cell clusters.

Cell cluster 1

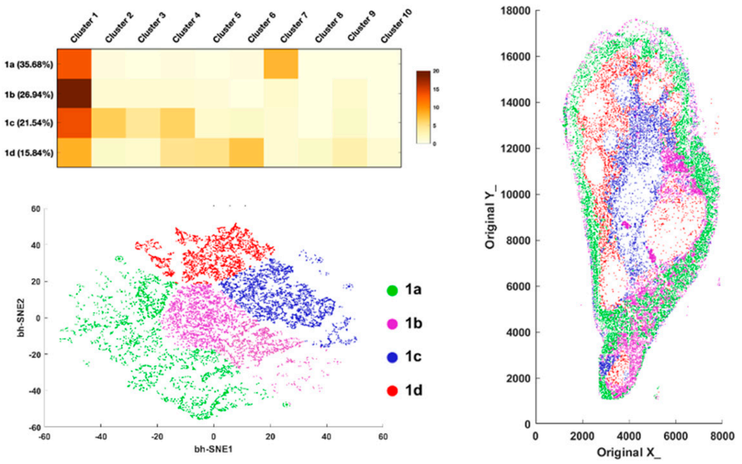

Cell cluster 2

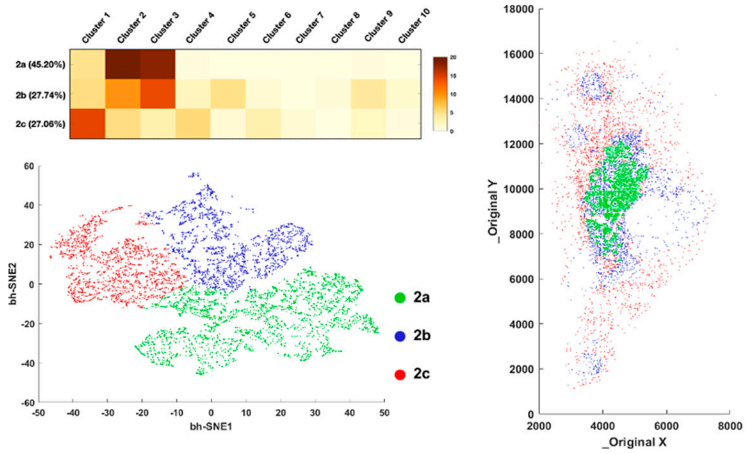

Cell cluster 3

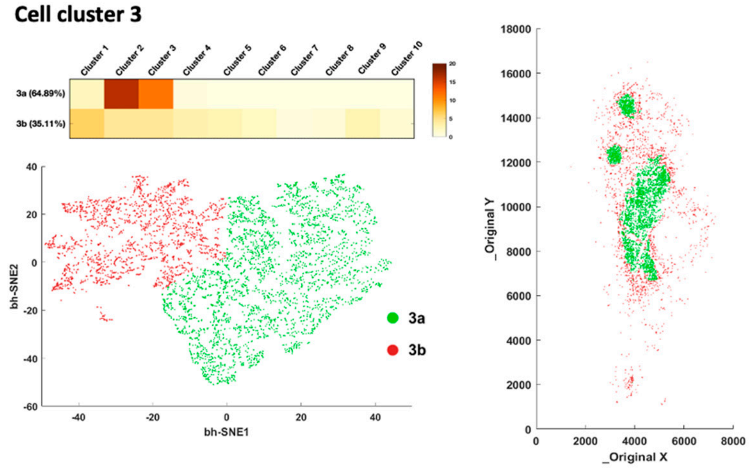

Cell cluster 4

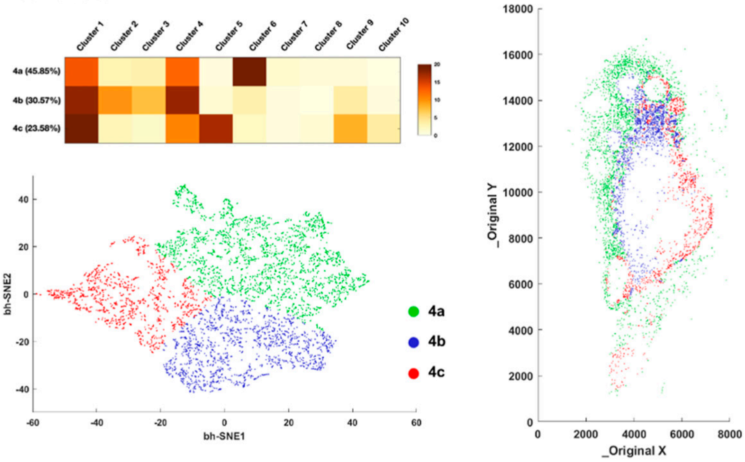

Cell cluster 5

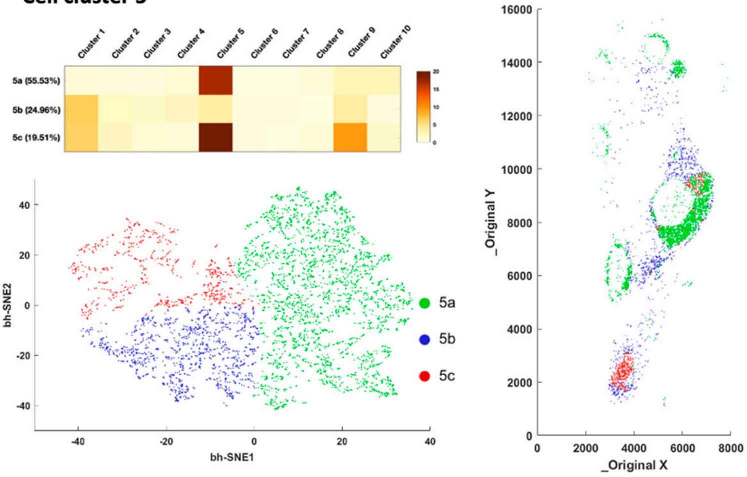

Cell cluster 6

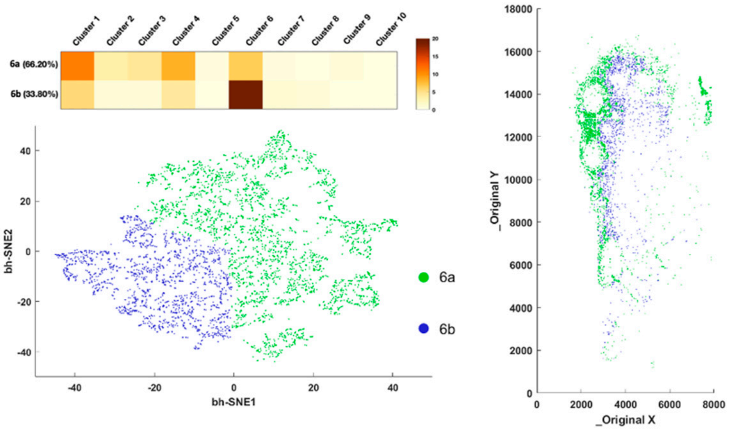

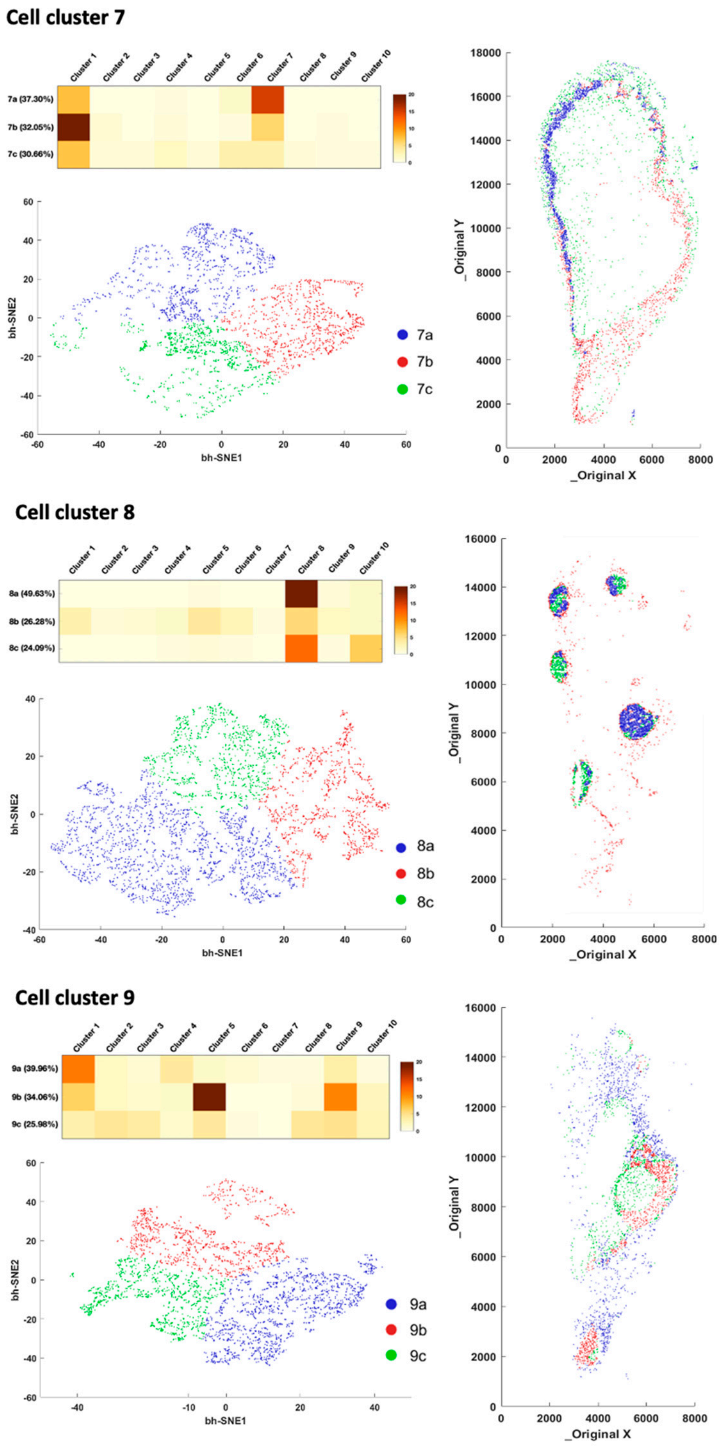

Figure S3. Further classification of the cells in varied microenvironment. For each cell cluster, based on their neighbor cells from different clusters (upper left), the individual cells in the every cell cluster are further partitioned into distinct subclusters (bottom left). The panels on the right display the anatomical locations of each cell from the different subclusters.
